# Supplementary material for: The Impact of Strength Changes on Active Function Following Botulinum Neurotoxin-A (BoNT-A): A Systematic Review
Source: Toxins (Basel). 2025 Jul 23;17(8):362. doi: 10.3390/toxins17080362 (PMC12390172; doi:10.3390/toxins17080362)
Supplement: Supplementary file 1 [file toxins-17-00362-s001.zip › toxins-3699136-supplementary/toxins-3699136 Supplementary File 1 - Round 2 Revised.pdf]

Supplementary File 1. Extended version of strength, and active function (i.e., activity), participation, or quality of life outcomes from studies included in the analysis (n = 17)[#].

Table S1.1 Upper Limb activity and strength outcomes of the muscle injected (i.e., agonist) and the opposing muscle (i.e., antagonist) (n = 7)[#].

	**Timepoint**	**≤6/52 weeks**			**>6/52 weeks to ≤3/12 months**			**>3 to ≤12/12 months**		
					**STRENGTH**					
		**Stronger**	**No change**	**Weaker**	**Stronger**	**No change**	**Weaker**	**Stronger**	**No change**	**Weaker**
					UPPER LIMB AGONIST STRENGTH					
ACTIVE FUNCTION	**Improved**		FF/Grip [45] $^{A}_{\dagger,6w}$ $^{B}_{6w}$<br>EF/Grip [47] $^{A}_{\dagger}$<br>EF/WF [46] $^{m}_{§}$							WF [49] ^{RUL}
	**No change**	Global [41] $^{B}_{G1, G2}$	FF/Grip [45] $^{A}_{\dagger, 2w}$$^{B}_{2w}$<br>FF/Grip [45]$^{Q}_{2w}$$^{Q}_{6w}$<br>EF/WF [46] $^{m}_{\infty}$		Global [41] $^{B}_{G1, G2}$	Grip [44] $^{B}_{E/C, E+C}$$^{GAS}_{E/C}$			Grip [43] $^{B}_{E/C, E+C}$	WF [49] $^{N9, N60, FUL}$
	**Worse**									
					UPPER LIMB ANTAGONIST STRENGTH					
	**Improved**	EE [46] $^{m}_{§}$	FE [45] $^{A}_{\dagger 6w}$$^{B}_{6w}$<br>EE [47] $^{A}_{\dagger}$<br>WE [46] $^{m}_{§}$					WE/FE [49] ^{RUL}		
	**No change**		FE [45] $^{A}_{\dagger,2w}$ $^{B}_{2w}$<br>FE [45] $^{Q}_{2w}$$^{Q}_{6w}$<br>EE/WE [46] $^{m}_{\infty}$					WE/FE [49] $^{N9, N60, FUL}$		
	**Worse**									

§—Subacute group; ∞—Chronic group; † – Total Score; A – Action Research Arm Test; B – Box and Blocks Test; C—Control Group; E—Experimental Group; EE – Elbow Extensors; EF – Elbow Flexors; F-UL - Functional Independence Measure - Upper Limb; GAS – Goal Attainment Scale; EF – Elbow Flexors; FE – Finger Extensors; FF – Finger Flexors; G1 – Group 1; G2 – Group 2; m – Modified Barthel Index; N60– Nine Hole Peg Test tested in 60 seconds; N9 – Nine Hole Peg Test tested on scale 0-9; Q – Quick DASH; RUL – Rivermead Motor Assessment: Upper Limb; w – weeks; WE – Wrist Extensors; WF – Wrist Flexors.

[#]Significance was reported as $p \leq 0.05$ unless otherwise stated.

Table S1.2 Relationship between upper limb strength (i.e., agonist or antagonist) outcomes and the corresponding change in active function (i.e., activity) (n = 7).

	All Time Frames			≤6/52 weeks			>6/52 weeks to ≤3/12 months			>3 – ≤12/12 months		
	STRENGTH											
	Improved n (%)	No Change n (%)	Worse n (%)	Improved n (%)	No Change n (%)	Worse n (%)	Improved n (%)	No Change n (%)	Worse n (%)	Improved n (%)	No Change n (%)	Worse n (%)
ACTIVE FUNCTION	UPPER LIMB AGONIST											
Improved n (%)	0 (0)	8 (31)	1 (25)	0 (0)	8 (44)	0 (0)	0 (0)	0 (0)	0 (0)	0 (0)	0 (0)	1 (25)
No Change n (%)	4 (100)	18 (69)	3 (75)	2 (100)	10 (56)	0 (0)	2 (100)	5 (100)	0 (0)	0 (0)	3 (100)	3 (75)
Worse n (%)	0 (0)	0 (0)	0 (0)	0 (0)	0 (0)	0 (0)	0 (0)	0 (0)	0 (0)	0 (0)	0 (0)	0 (0)
Total	4 (100)	26 (100)	4 (100)	2 (100)	18 (100)	0 (0)	2 (100)	5 (100)	0 (0)	0 (0)	3 (100)	4 (100)
	UPPER LIMB ANTAGONIST											
Improved n (%)	3 (33)	4 (40)	0 (0)	1 (100)	4 (40)	0 (0)	0 (0)	0 (0)	0 (0)	2 (25)	0 (0)	0 (0)
No Change n (%)	6 (67)	6 (60)	0 (0)	0 (0)	6 (60)	0 (0)	0 (0)	0 (0)	0 (0)	6 (75)	0 (0)	0 (0)
Worse n (%)	0 (0)	0 (0)	0 (0)	0 (0)	0 (0)	0 (0)	0 (0)	0 (0)	0 (0)	0 (0)	0 (0)	0 (0)
Total	9 (100)	10 (100)	0 (0)	1 (100)	10 (100)	0 (0)	0 (0)	0 (0)	0 (0)	8 (100)	0 (0)	0 (0)

n – number of outcomes; % - percentage

Table S1.3 Lower limb activity and strength outcomes of the muscle injected (i.e., agonist) and opposing muscle (i.e., antagonist) (n = 10)[#].

Timepoint	≤6/52 weeks			>6/52 weeks to ≤3/12 months			>3 to ≤12/12 months		
**Strength**	**Stronger**	**No change**	**Weaker**	**Stronger**	**No change**	**Weaker**	**Stronger**	**No change**	**Weaker**
**ACTIVE FUNCTION** — LOWER LIMB AGONIST STRENGTH									
**Improved**		Global [38] [a]G1[c]G1,G2<br>PF [48] [aº,x^,r-L+T]	KE [£,¢] [35] [ai, Sa]<br>PF [38] [a]G1[c]G1, G2<br>PF [MRC/QMA] [39] [a]		PF/Global [38] [a]G1[c]G1<br>PF [48] [aº,x^,r-L+T]			PF [MRC/QMA] [39] [a]<br>PF [48] [aº,x^,r-L+T]	
**No change**		Global [38] [a]G2<br>PF [48] [a^,bº,xš]	KE [£,¢] [35] [c]<br>KE [¥,€,Ÿ,Ý,Ž] [42] [a,b,c,e,Sa,Sd]<br>PF [38] [a]G2		HAd/PF [40] [a,b]<br>PF [36] [f,x]<br>PF [48] [a^,bº,xš]<br>Global [38] [a]G2[c]G2	PF [38] [a]G2[c]G2		PF [48] [bº,a^,xš]<br>PF [36] [a,x,f,l]<br>PF [MRC/QMA] [39] [b,e]	
**Worse**									
**ACTIVE FUNCTION** — LOWER LIMB ANTAGONIST STRENGTH									
**Improved**	DF [38] [a]G1[c]G1, G2	DF [34] [a,d]G1,2-20 days<br>DF‡ [37] [a]Ca[c] Ta,Ca<br>DF [48] [aº,x^,r-L+T]<br>HAb/DF L,R [50] [aº]		DF [38] [a]G1[c]G1	DF [34] [a,d]G1,G2-90 days<br>DF‡ [37] [a]Ca[c]Ta,Ca<br>DF [48] [aºx^, r-L+T]			DF [48] [aº,x^,r-L+T]<br>HAb/DF L,R [50] [aº]	
**No change**	KF [¥,€,Ý] [42] [a,b,c,e,Sa,Sd]<br>DF [38] [a]G2	KF [Ÿ,Ž] [42] [a,b,c,e,Sa,Sd]<br>DF‡ [37] [a]Ta[a,c]St[x]Ta,Ca,St<br>DF [34] [a,d]G1, G2-10 days<br>DF [48] [a^,bº,xš]<br>HAb/DF L,R [50] [xº,x^,bº,r-L+T]			DF‡ [37] [a]Ta,St[c]St [x]Ta,Ca,St<br>DF [36] [f,x]<br>DF [38] [a]G2[c]G2<br>DF [48] [a^,bº,xš]<br>HAb/DF L,R [50] [xº,x^,aº,bº,r-L+T]			DF [36] [a,x,f,l]<br>DF [48] [bº,a^,xš]<br>HAb/DF L,R [50] [xº,x^,bº,r-L+T]	
**Worse**									

‡ – p Value Set at $p < 0.02$; ^ – barefoot; °/s – degrees per second; ¢ – Hip Flexion 90◦ as reported by the author with p-Value Set at $p < 0.01$; £ – Strength tested at Hip Flexion 0◦; ¥ – Strength outcome measure was maximal voluntary contraction of isometric torque at 40◦ (Nm); € – Strength tested through maximal voluntary contraction concentric torque 90◦/s (Nm); a – Ten Metre Walk Test Comfortable; ai – Gait Velocity; b –Ten Metre Walk Test Fast; c – Six Minute Walk Test; C—Control Group; d – 2-Minute Walk Test; DF – Dorsiflexors; e – Timed Up and Go Test; E—Experimental Group; f – Functional Walking Category; G1 – Group 1; G2 – Group 2; HAb – Hip Abduction; HAd – Hip Adduction; is – Isometric; KE – Knee Extensors; KF – Knee Flexors; l – ABILOCO Functional Locomotion Outcome measure; L – Left; r-L+T – Rivermead Motor Assessment: Leg and Trunk; MRC – Medical Research Council Scale; º – With Usual Aid; PF – Plantarflexors; QMA – Quantitative Muscle Assessment; R – Right; š – With Shoes and aids; Sa – Stair Climb Ascend; Sd – Stair Climb Descend; St – Stretching Group; Ta – Taping Group; x – Functional Ambulation Category; Ý – Strength outcome measure was maximal voluntary contraction of isometric torque at 60◦ (Nm); Ÿ – Strength outcome measure was maximal voluntary contraction of concentric torque 30◦/s (Nm); Ž – Strength outcome measure was maximal voluntary contraction of concentric torque 60◦/s (Nm).

[#]Significance was reported as $p \leq 0.05$ unless otherwise stated.

Table S1.4 Relationship between lower limb strength (i.e., agonist or antagonist) outcomes and the corresponding change in active function (i.e., activity) (n = 10)[#].

		All Time Frames			≤6/52 weeks			>6/52 weeks to ≤3/12 months			>3 – ≤12/12 months		
		STRENGTH											
		Improved n (%)	No Change n (%)	Worse n (%)	Improved n (%)	No Change n (%)	Worse n (%)	Improved n (%)	No Change n (%)	Worse n (%)	Improved n (%)	No Change n (%)	Worse n (%)
ACTIVE FUNCTION		LOWER LIMB AGONIST STRENGTH											
	Improved n (%)	0 (0)	18 (41)	7 (17)	0 (0)	6 (60)	7 (18)	0 (0)	7 (39)	0 (0)	0 (0)	5 (31)	0 (0)
	No Change n (%)	0 (0)	26 (59)	34 (83)	0 (0)	4 (40)	32 (82)	0 (0)	11 (61)	2 (100)	0 (0)	11 (69)	0 (0)
	Worse n (%)	0 (0)	0 (0)	0 (0)	0 (0)	0 (0)	0 (0)	0 (0)	0 (0)	0 (0)	0 (0)	0 (0)	0 (0)
	Total	0 (0)	44 (100)	41 (100)	0 (0)	10 (100)	39 (100)	0 (0)	18 (100)	2 (100)	0 (0)	16 (100)	0 (0)
		LOWER LIMB ANTAGONIST STRENGTH											
	Improved n (%)	5 (21)	31 (24)	0 (0)	3 (14)	14 (25)	0 (0)	2 (100)	10 (23)	0 (0)	0 (0)	7 (23)	0 (0)
	No Change n (%)	19 (79)	97 (76)	0 (0)	19 (86)	41 (75)	0 (0)	0 (0)	33 (77)	0 (0)	0 (0)	23 (77)	0 (0)
	Worse n (%)	0 (0)	0 (0)	0 (0)	0 (0)	0 (0)	0 (0)	0 (0)	0 (0)	0 (0)	0 (0)	0 (0)	0 (0)
	Total	24 (100)	128 (100)	0 (0)	22 (100)	55 (100)	0 (0)	2 (100)	43 (100)	0 (0)	0 (0)	30 (100)	0 (0)

n – number of outcomes; % - percentage

Table S1.5 Strength (i.e., upper and lower limb agonist and antagonist) and participation and QoL outcomes (n = 4)[#].

	**Timepoint**	**≤6/52 weeks**			**>6/52 weeks to ≤3/12 months**			**>3 to ≤12/12 months**		
**PARTICIPATION AND QOL**	**Strength**	**Stronger**	**No Change**	**Weaker**	**Stronger**	**No Change**	**Weaker**	**Improved**	**No Change**	**Weaker**
		UPPER LIMB AGONIST STRENGTH								
	**Improved**	Global [41] $^{h}_{G1,G2}$			Global [41] $^{h}_{G1, G2}$					
	**No change**					Grip [44] $^{y,z}_{E/C}$ $^{y}_{E+C}$			Grip [43] $^{y}_{E/C}$ $^{y}_{E+C}$	
	**Worse**									
		LOWER LIMB AGONIST AND *ANTAGONIST* STRENGTH								
	**Improved**									
	**No change**								PF/*DF* [36] ^{n,u,v}	
	**Worse**									

Antagonist result in italics; C—Control Group; DF – Dorsiflexors; E—Experimental Group; G1–Group 1; G2–Group 2; h – Stroke Impact Scale; n – SATISPART - Satisfaction – Stroke Questionnaire; PF – Plantarflexors; u – SF-36 - Physical Health; v – SF-36 - Mental Health; y - QOL-EQ-5D OH – Quality of Life Euroqual-5D Overall Health; z – QOL- EQ-5D SC – Quality of Life Euroqual – 5D Self-care.

[#]Significance was reported as p ≤ 0.05 unless otherwise stated.

Table S1.6 Relationship between strength (i.e., upper and lower limb agonist and antagonist) outcomes and the corresponding active function, participation and QoL outcomes (n = 4).

		All Time Frames			≤6/52 weeks			>6/52 weeks to ≤3/12 months			>3 – ≤12/12 months		
		STRENGTH											
		Improved n (%)	No Change n (%)	Worse n (%)	Improved n (%)	No Change n (%)	Worse n (%)	Improved n (%)	No Change n (%)	Worse n (%)	Improved n (%)	No Change n (%)	Worse n (%)
ACTIVE FUNCTION		UPPER LIMB AGONIST STRENGTH											
	Improved n (%)	4 (100)	0 (0)	0 (0)	2 (100)	0 (0)	0 (0)	2 (100)	0 (0)	0 (0)	0 (0)	0 (0)	0 (0)
	No Change n (%)	0 (0)	8 (100)	0 (0)	0 (0)	0 (0)	0 (0)	0 (0)	5 (100)	0 (0)	0 (0)	3 (100)	0 (0)
	Worse n (%)	0 (0)	0 (0)	0 (0)	0 (0)	0 (0)	0 (0)	0 (0)	0 (0)	0 (0)	0 (0)	0 (0)	0 (0)
	Total	4 (100)	8 (100)	0 (0)	2 (100)	0 (0)	0 (0)	2 (100)	5 (100)	0 (0)	0 (0)	3 (100)	0 (0)
		LOWER LIMB AGONIST AND *ANTAGONIST* STRENGTH											
	Improved n (%)	0 (0)	0 (0)	0 (0)	0 (0)	0 (0)	0 (0)	0 (0)	0 (0)	0 (0)	0 (0)	0 (0)	0 (0)
	No Change n (%)	0 (0)	6 (100)	0 (0)	0 (0)	0 (0)	0 (0)	0 (0)	0 (0)	0 (0)	0 (0)	6 (100)	0 (0)
	Worse n (%)	0 (0)	0 (0)	0 (0)	0 (0)	0 (0)	0 (0)	0 (0)	0 (0)	0 (0)	0 (0)	0 (0)	0 (0)
	Total	0 (0)	6 (100)	0 (0)	0 (0)	0 (0)	0 (0)	0 (0)	0 (0)	0 (0)	0 (0)	6 (100)	0 (0)

n – number of outcomes; % - percentage
