# Supplementary material for: The Impact of Strength Changes on Active Function Following Botulinum Neurotoxin-A (BoNT-A): A Systematic Review
Source: Toxins (Basel). 2025 Jul 23;17(8):362. doi: 10.3390/toxins17080362 (PMC12390172; doi:10.3390/toxins17080362)
Supplement: Supplementary file 1 [file toxins-17-00362-s001.zip › toxins-3699136-supplementary/toxins-3699136 Supplementary File 2 - Round 2 Revised.pdf]

Supplementary File 2. Detailed Activity outcome data from articles (n = 17)[#].

Study	Outcome Measure (Unit of Measure)	Group Details	Pre-injection Mean ± (SD) or Median [IQR] (95% CI)	Post-injection Mean ± SD (95% CI) Median [IQR] (P25, P75)	Within-Group Difference, Mean ± SD	Within-group Difference	Timepoints
Baricich 2019 [34] (n=30)	10MWT (m/s) *Mean (SD)	G1: 1 x ES of Injected Muscles + 5 x ES of TA (n=15)	0.6 (0.4)	0.6 (0.4) T1<br>0.7 (0.4) T2<br>0.8 (0.5) T3	NR<br>NR<br>NR	NS<br>+, SS<br>+, SS	T1 = 10/7<br>T2 = 20/7<br>T3 = 90/7
		G2: 1 x ES of Injected Muscles (n=15)	0.6 (0.3)	0.7 (0.3) T1<br>0.7 (0.3) T2<br>0.8 (0.3) T3	NR<br>NR<br>NR	NS<br>+, SS<br>+, SS	
	2MWT (m) *Mean (SD)	G1: 1 x ES of Injected Muscles + 5 x ES of TA (n=15)	63.5 (42)	71 (43) T1<br>73 (44) T2<br>76 (49) T3	NR<br>NR<br>NR	NS<br>+, SS<br>+, SS	
		G2: 1 x ES of Injected Muscles (n=15)	76 (32)	82 (31) T1<br>86 (32) T2<br>90 (33) T3	NR<br>NR<br>NR	NS<br>+, SS<br>+, SS	
Bernuz 2012 [35] (n=15)	6MWT (m) *Mean ± SD	Pre-Post BoNT-A	331 ± 146	332 ± 143	NR	NS	T1 = 4-6/52
	Timed stairs climbing (s) *Mean ± SD		39.6 ± 38.0	30.1 ± 14.0	NR	+, SS	
	Gait velocity (cm/s) 3DMA 15m walkway *Mean ± SD		56.80 ± 31.20	67.50 ± 34.30	NR	+, SS	
Bollens 2013 [36] (n=16)	FWC (0-6) * Median [IQR]	BoNT-A alone (n=8)	5 [4-5] *ƒ*	5 [5-5] T1 *ƒ*<br>5 [5-5] T2 *ƒ*	NR<br>NR	NS<br>NS	T1 = 2/12<br>T2 = 6/12
	FAC (0-5) * Median [IQR]		4 [4-4] *ƒ*	4 [4-4] T1 *ƒ*<br>4 [4-4] T2 *ƒ*	NR<br>NR	NS<br>NS	
	10MWT (m/s) * Mean ± SD		0.62 ± 0.41	0.65 ± 0.39 T1<br>0.64 ± 0.39 T2	NR<br>NR	NR<br>NS	
	ABILOCO, logits *Mean ± SD		1.79 ± 2.65	2.69 ± 2.53 T1<br>2.44 ± 2.40 T2	NR<br>NR	NR<br>NS	
Carda 2011 [37] (n=69)	10MWT (s) *Mean (SD)	Taping (n=24)	28.2 (22.4)	26.2 (23.0) T1 (n=23)<br>24.1 (15.0) T2 (n=23)	NR<br>NR	NS<br>NS	T1 = ~3/52<br>T2 = 3/12
		Casting (n=27)	25.4 (16.0)	21.5 (14.3) T1 (n=26)<br>18.4 (11.6) T2 (n=23)	NR<br>NR	+, SS<br>+, SS	
		Stretching (n=18)	27.3 (22.9)	26.4 (25.0) T1 (n=18)<br>26.9 (21.9) T2 (n=18)	NR<br>NR	NS<br>NS	
	6MWT (m) *Mean (SD)	Taping (n=24)	176 (99)	204 (108) T1 (n=23)<br>211 (107) T2 (n=23)	NR<br>NR	+, SS<br>+, SS	
		Casting (n=27)	192 (99)	215 (114) T1 (n=26)<br>246 (125) T2 (n=23)	NR<br>NR	+, SS<br>+, SS	
		Stretching (n=18)	196 (81)	206 (77) T1 (n=18)<br>197 (84) T2 (n=18)	NR<br>NR	NS<br>NS	
	FAC (0-5) *Mean (SD)	Taping (n=24)	4 (1)	4 (1) T1 (n=23)<br>4 (1) T2 (n=23)	NR<br>NR	NS<br>NS	
		Casting	4 (1)	4 (1) T1 (n=26)	NR	NS	

Supplementary File 2. Detailed Activity outcome data from articles (n = 17)[#].

		(n=27)		5 (1) T2 (n=23)	NR	NS	
		Stretching (n=18)	4 (1)	4 (1) T1 (n=18)<br>4 (1) T2 (n=18)	NR<br>NR	NS<br>NS	
Cinone 2019 [38] (n=25)	10MWT (m/s) *Mean (SD)	G1: BoNT-A + 4/52 Isokinetic Training (n=12)	0.62 (0.22)	0.69 (0.26) T1<br>0.65 (0.24) T2	- 2.37 (T0-T1)<br>- 2.21 (T0-T2)	+, SS<br>+, SS	T1 = 5/52<br>T2 = 8/52
		G2: BoNT-A alone (n=13)	0.61 (0.18)	0.64 (0.21) T1<br>0.62 (0.12) T2	NR<br>NR	NS<br>NS	
	6MWT (m) *Mean (SD)	G1: BoNT-A + 4/52 Isokinetic Training (n=12)	221 (90)	254 (9) T1<br>236 (87) T2	- 2 (T0-T1)<br>- 2 (T0-T2)	+, SS<br>+, SS	
		G2: BoNT-A alone (n=13)	217 (90)	236 (92) T1<br>221 (90) T2	NR<br>NR	+, SS<br>NS	
de Niet 2015 [39] (n=15)	10MWT (Comfortable Gait Velocity (m/s) *Mean (SD) [95% CI] (m/s)	Pre-Post BoNT-A	0.90 (0.18) [0.80–0.97]	0.98 (0.22) [0.87–1.09] T1<br>1.01 (0.19) [0.91–1.11] T2	+ 9 % (T0-T1)<br>+ 12 % (T0-T2)	+, SS<br>+, SS	T1 = 4/52<br>T2 = 18/52
	10MWT (Maximum Gait Velocity) (m/s) *Mean (SD) [95% CI] (m/s)		1.33 (0.34) [1.16–1.50]	1.33 (0.33) [1.17–1.50] T1<br>1.33 (0.37) [1.14–1.51] T2	NR<br>NR	NR<br>NS	
	TUG *Mean (SD) [95% CI] (s)		10.4 (2.8) [8.9–11.8]	10.5 (2.3) [9.3–11.7] T1<br>10.9 (2.5) [9.7–12.2] T2	NR<br>NR	NR<br>NS	
Diniz de Lima 2021 [40] (n=55)	10MWT Maximal Gait Velocity (m/s) *Mean ± SD	Treatment (BoNT-A)	1.02 ± 0.57 (n=54)	1.01 ± 0.59 (n=52)	NR	NS	T1 = 8/52
	10MWT Self-Selected Gait Velocity (m/s) *Mean ± SD	Cross over trial	0.77 ± 0.38 (n=54)	0.74 ± 0.37 (n=52)	NR	NS	
Giray 2020 [41] (n=20)	BBT (0-60s) º *Median ± IQR	G1: Lycra sleeve + Rehab (n=10)	0 [0–6]	0 [0–11] T1<br>0 [0–9] T2	NR<br>NR	NS<br>NS	T1 = 3/52<br>T2 = 3/12
		G2: Rehab only (n=10)	1 [0–16]	1 [0–10] T1<br>0 [0–19] T2	NR<br>NR	NS<br>NS	
Hameau 2014 [42] (n=14)	6MWT (m) *Mean ± SD	Pre-Post BoNT-A	342 (112)	339 (109)	NR	NS	T1 = 4/52
	10MWT - Maximal Velocity (s) *Mean ± SD		9.4 (4.0)	9.3 (3.8)	NR	NS	
	10MWT - Spontaneous Velocity (s) *Mean ± SD		13.0 (4.7)	13.2 (4.8)	NR	NS	
	TUG (s) *Mean ± SD		12.1 (4.0)	11.9 (4.3)	NR	NS	
	Time to Ascend Stairs (x4)(s) *Mean ± SD		5.0 (1.3)	5.1 (1.8)	NR	NS	
	Time to Descend Stairs (x4)(s) *Mean ± SD		4.6 (1.9)	4.6 (1.7)	NR	NS	
Lannin 2020 [44] (n=139)	BBT, Blocks/s *Mean (SD)	E: BoNT-A + evidence-based movement training (n=69)	0 (0)	0 (0) (n=67)	0 (0)	NS	T1 = 3/12
		C: BoNT-A + usual care (n=71)	0 (0)	0 (0) (n=71)	0 (0)	NS	
		E+C (n=138)	2 (1)	2 (1)	0 (0)	NS	
	GAS (T-score) *Mean (SD)	E: BoNT-A + evidence-based movement training (n=67)	N/A	43 (12)	NA	NS	
		C: BoNT-A + usual care (n=71)	N/A	41 (12)	NA	NS	

Supplementary File 2. Detailed Activity outcome data from articles (n = 17)#.

Lannin 2022 [43]<br>(n=140)	BBT, Blocks/s (n=130)<br>*Mean (SD)	E: BoNT-A + evidence-based movement training (n=69)	0 (0)	0 (0) (n=64)	0 (0)	NS	T1 = 12/12
		C: BoNT-A + usual care (n=71)	0 (0)	0 (0) (n=66)	0 (0)	NS	
		E+C (n=130)	0 (0)	0 (0)	MD 0.01 [95% CI 0.00 to 0.02]	NS	
	GAS (T-score)<br>*Mean (SD)	E: BoNT-A + evidence-based movement training (n=69)	N/A	41 (14) (n=65)	NA	NR	
		C: BoNT-A + usual care (n=71)	N/A	41 (13) (n=68)	NA	NR	
Lee 2018 [45]<br>(n=15)	BBT (Blocks in 60s)<br>*Mean ± SD	Pre-Post BoNT-A	3.0 ± 4	4 ± 5 T1<br>5 ± 5 T2	NR<br>NR	NS<br>NS<br>+, SS	T1 = 2/52<br>T2 = 6/52
	ARAT – Total (0-57)<br>*Mean ± SD		11 ± 8	11 ± 8 T1<br>13 ± 8 T2	NR<br>NR	NS<br>+, SS<br>+, SS	
	QDASH (0-100)<br>*Mean ± SD		57 ± 17	55 ± 15 T1<br>54 ± 17 T2	NR<br>NR	NS<br>NS<br>NS	
Lim 2016 [46]<br>(n=18)	MBI (0-20)<br>*Mean ± SD	Subacute (n=9)	35 ± 34	52 ± 32	NR	+, SS	T1 = 4/52
		Chronic (n=9)	76 ± 25	77 ± 25	NR	NS	
Pandyan 2002 [47]<br>(n=14)	ARAT (0-56) *Mean ± SD	Pre-Post BoNT-A	4 ± 2 *ƒ*	5 ± 4 *ƒ*	z = - 2 *ƒ*	+, SS	T1 = 4/52
Rousseaux 2002 [49]<br>(n=20)	RMA–Arm (0-15)<br>*Mean (SD)	Pre-Post BoNT-A	5 (2)	5 (3) T1<br>5 (3) T2<br>5 (3) T3	1 (1) T1<br>1 (1) T2<br>1 (1) T3	NR<br>NR<br>+, SS	T1 = 2/52<br>T2 = 2/12<br>T3 = 5/12
	Nine-Hole Peg Test (0-9)<br>*Mean (SD)		2 (3)	2 (3) T1<br>2 (3) T2<br>2 (3) T3	0 (2) T1<br>0 (2) T2<br>0 (2) T3	NR<br>NR<br>NS	
	Nine-Hole Peg Test 0/60s<br>*Mean (SD)		59 (2)	60 (1) T1<br>59 (5) T2<br>58 (7) T3	0 (3) T1<br>1 (6) T2<br>1 (8) T3	NR<br>NR<br>NS	
	FIM-UL (0-35) (feeding, grooming, bathing, dressing UL, dressing LL) *Mean (SD)		28 (7)	28 (6) T1<br>28 (6) T2<br>28 (6) T3	0 (0) T1<br>0 (1) T2<br>0 (1) T3	NR<br>NR<br>NS	
Rousseaux 2005 [48]<br>(n=47)	Gait velocity: 10 m (m/s)<br>*Mean (SD)	Comfortable with usual aid — Pre-Post BoNT-A	0.48 (0.29)	0.52 (0.31) T1<br>0.51 (0.30) T2<br>0.54 (0.31) T3	0.03 (0.08) T1<br>0.02 (0.08) T2<br>0.05 (0.10) T3	+, SS<br>+, SS<br>+, SS	T1 = 2/52<br>T2 = 2/12<br>T3 = 5/12
		Rapid with aid — Pre-Post BoNT-A	0.63 (0.43)	0.66 (0.45) T1<br>0.66 (0.42) T2<br>0.66 (0.41) T3	0.03 (0.12) T1<br>0.03 (0.11) T2<br>0.03 (0.15) T3	NS<br>NS<br>NS	
		Comfortable Barefoot, no aid — Pre-Post BoNT-A	0.65 (0.24)	0.70 (0.24) T1<br>0.68 (0.24) T2<br>0.68 (0.23) T3	0.05 (0.11) T1<br>0.03 (0.09) T2<br>0.04 (0.09) T3	NS<br>NS<br>NS	
	FAC (0-5)<br>*Mean (SD)	Barefoot — Pre-Post BoNT-A	2.53 (1.62)	2.85 (1.51) T1<br>2.87 (1.44) T2<br>2.79 (1.51) T3	0.32 (0.52) T1<br>0.34 (0.61) T2<br>0.26 (0.56) T3	+, SS<br>+, SS<br>+, SS	
		With shoes + aids — Pre-Post BoNT-A	4.47 (0. 54)	4.51 (0.55) T1<br>4.50 (0.54) T2<br>4.50 (0.55) T3	0.04 (0.18) T1<br>0.03 (0.16) T2<br>0.03 (0.19) T3	NS<br>NS<br>NS	

Supplementary File 2. Detailed Activity outcome data from articles (n = 17)[#].

	RMA: Leg and Trunk (0-10)<br>*Mean (SD)	Pre-Post BoNT-A	5 (2)	6 (2) T1<br>6 (2) T2<br>6 (2) T3	0 (1) T1<br>0 (1) T2<br>0 (1) T3	+, SS<br>+, SS<br>+, SS	
Rousseaux 2007<br>[50]<br>(n=15)	FAC - Barefoot (0-5)<br>*Median	Pre-Post BoNT-A	4	4 T1<br>4 T2<br>4 T3	NR T1<br>NR T2<br>NR T3	NS<br>NS<br>NS	T1 = 2-3/52<br>T2 = 2-3/12<br>T3 = 5/12
	FAC - With usual aid (0-5)<br>*Median		5	5 T1<br>5 T2<br>5 T3	NR T1<br>NR T2<br>NR T3	NS<br>NS<br>NS	
	RMA: Leg and Trunk (0-16)<br>*Median		12	12 T1<br>12 T2<br>9 T3	NR T1<br>NR T2<br>NR T3	NS<br>NS<br>NS	
	Gait Velocity 10m (m/s)<br>Comfortable with usual aid<br>10m *Median		0.71	0.77 T1<br>0.71 T2<br>0.71 T3	NR T1<br>NR T2<br>NR T3	+, SS<br>NS<br>+SS	
	Gait Velocity 10m (m/s)<br>Rapid with usual aid<br>*Median		1.00	1.11 T1<br>1.00 T2<br>1.00 T3	NR T1<br>NR T2<br>NR T3	NS<br>NS<br>NS	

+ – Significantly improved; 10MWT – 10 Metre Walk Test; 2MWT – Two Minute Walk Test; 6MWT – Six Minute Walk Test; ABILOCO – Measure of locomotion ability for adults with lower limb impairments; ARAT – Action Research Arm Test; BBT – Box and Block Test; BoNT-A – Botulinum Neuro Toxin-A; CI - Confidence Interval; cm/s – centre metres per second; ES – E-Stims; *f* – Calculated By Authors Based On Supplied Data; FAC – Functional Ambulation Category; FIM – Functional Independence Measure – Upper Limb; FWC – Functional Walking Category; G1 – Group 1; G2 – Group 2; GAS – Goal Attainment Scale; IQR – Interquartile Range; m – metres; m/s – metres per second; MD – Mean Difference; NR – Not Reported; NS – Non-Significant; º – Number of blocks transferred in 60 seconds on the BBT; QDASH – Quick Disabilities Of Arm, Shoulder, and Hand; RMA – Rivermead Motor Assessment; s – seconds; SD – Standard Deviation; SF-36 – Short Form Survey 36 Item Scale; SS – Statistically Significant; T1 – Timepoint 1; T2 – Timepoint 2; T3 – Timepoint 3; TA – Tibialis Anterior; TUG – Timed Up and Go Test; UL – Upper Limb; VAS –Visual Analogue Scale.

[#] Significance was reported as $p \leq 0.05$ unless otherwise stated.

Supplementary File 2. Detailed Activity outcome data from articles (n = 17)[#].
