# Supplementary material for: The Impact of Strength Changes on Active Function Following Botulinum Neurotoxin-A (BoNT-A): A Systematic Review
Source: Toxins (Basel). 2025 Jul 23;17(8):362. doi: 10.3390/toxins17080362 (PMC12390172; doi:10.3390/toxins17080362)
Supplement: Supplementary file 1 [file toxins-17-00362-s001.zip › toxins-3699136-supplementary/toxins-3699136 Supplementary File 3 - Round 2 Revised.pdf]

Supplementary File 3. Detailed data of Participation and QOL outcomes from articles (n = 4)[#].

Study	Outcome Measure (Unit of Measure)	Group Details/Design	Pre-Injection Mean ± (SD)	Post-Injection Mean ± SD	Within-Group Difference, Mean ± SD	Within-Group Change	Timepoints
PARTICIPATION OUTCOMES							
Bollens 2013 [36] (n=8)	SATISPART - Stroke, Logits *Mean ± SD	BoNT-A alone (n=8)	0.20 ± 0.83	0.39 ± 0.84 T1<br>0.31 ± 0.79 T2	NR<br>NR	NR<br>NS	T1 = 2/12<br>T2 = 6/12
QUALITY OF LIFE OUTCOMES							
Bollens 2013 [36] (n=8)	QOL-SF-36- PH (0-100) *Mean ± SD	BoNT-A alone (n=8)	34 ± 10	37 ± 7 T1<br>34 ± 12 T2	NR<br>NR	NR<br>NS	T1 = 2/12<br>T2 = 6/12
	QOL-SF-36 - MH (0-100) *Mean ± SD		34 ± 6	32 ± 8 T1<br>37 ± 13 T2	NR<br>NR	NR<br>NS	
Giray 2020 [41] (n=20)	Stroke Impact Scale (0-100) *Median [IQR]	G1: Lycra Sleeve + Rehab (n=10)	137 (122, 210)	208 (182, 227) T1<br>209 (176, 238) T2	NR<br>NR	+, SS<br>+, SS	T1 = 3/52<br>T2 = 3/12
		G2: Rehab Only (n=10)	128 (111, 143)	209 (184, 227) T1<br>192 (163, 224) T2	NR<br>NR	+, SS<br>+, SS	
Lannin 2020 [44] (n=139)	QOL-EQ-5D - Overall Health (0-100) *Mean (SD)	E: BoNT-A + Evidence-Based Movement Training	65 (21) (n=69)	64 (25) (n=67)	−1 (-11)	NS	T1 = 3/12
		C: BoNT-A + Usual Care (n=71)	62 (20)	63 (23)	2 (25)	NS	
		E + C (n=138)	63 (21)	63 (24)	0 (-4 – 5)	NS	
	QOL-EQ-5D - Self-Care (0-3) *Mean (SD)	E: BoNT-A + Evidence-Based Movement Training	2 (1) (n=69)	2 (1) (n=67)	0 (1)	NS	
		C: BoNT-A + Usual Care (n=71)	2 (1)	2 (1)	0 (0)	NS	
Lannin 2022 [43] (n=140)	QOL-EQ-5D - Overall Health (0-100) *Mean (SD)	E: BoNT-A + Evidence-Based Movement Training	65 (21) (n=69)	67 (23) T2 (n=65)	3 (24)	NS	T1 = 12/12
		C: BoNT-A + Usual Care	62 (20) (n=71)	64 (24) T2 (n=68)	3 (18)	NS	
		E + C (n=133)	63 (21)	66 (23)	MD 3 [95% CI -1.00 to 7.00]	NS	

+ – Significantly improved; BoNT-A – Botulinum Toxin A; C – Control; EQ-5D – Euroqual-5D Questionnaire; E – Experimental; G1 – Group 1; G2 – Group 2; MD – Mean Difference; NR – Not Reported; NS – Non-Significant; QOL SF 36 – PH – Quality of Life Short Form 36-Physical Health; QOL SF 36 – MH – Quality of Life Short Form 36-Mental Health; SS – Statistically Significant; T1 – Timepoint 1; T2 – Timepoint 2; T3 – Timepoint 3.

[#]Significance was reported as $p \leq 0.05$ unless otherwise stated.
