# Supplementary material for: The Impact of Strength Changes on Active Function Following Botulinum Neurotoxin-A (BoNT-A): A Systematic Review
Source: Toxins (Basel). 2025 Jul 23;17(8):362. doi: 10.3390/toxins17080362 (PMC12390172; doi:10.3390/toxins17080362)
Supplement: Supplementary file 1 [file toxins-17-00362-s001.zip › toxins-3699136-supplementary/toxins-3699136 Supplementary File 4 - Round 2 Revised.pdf]

Supplementary File 4. Methodological quality assessment for the modified Downs and Black checklist and PEDro scale (n = 17).

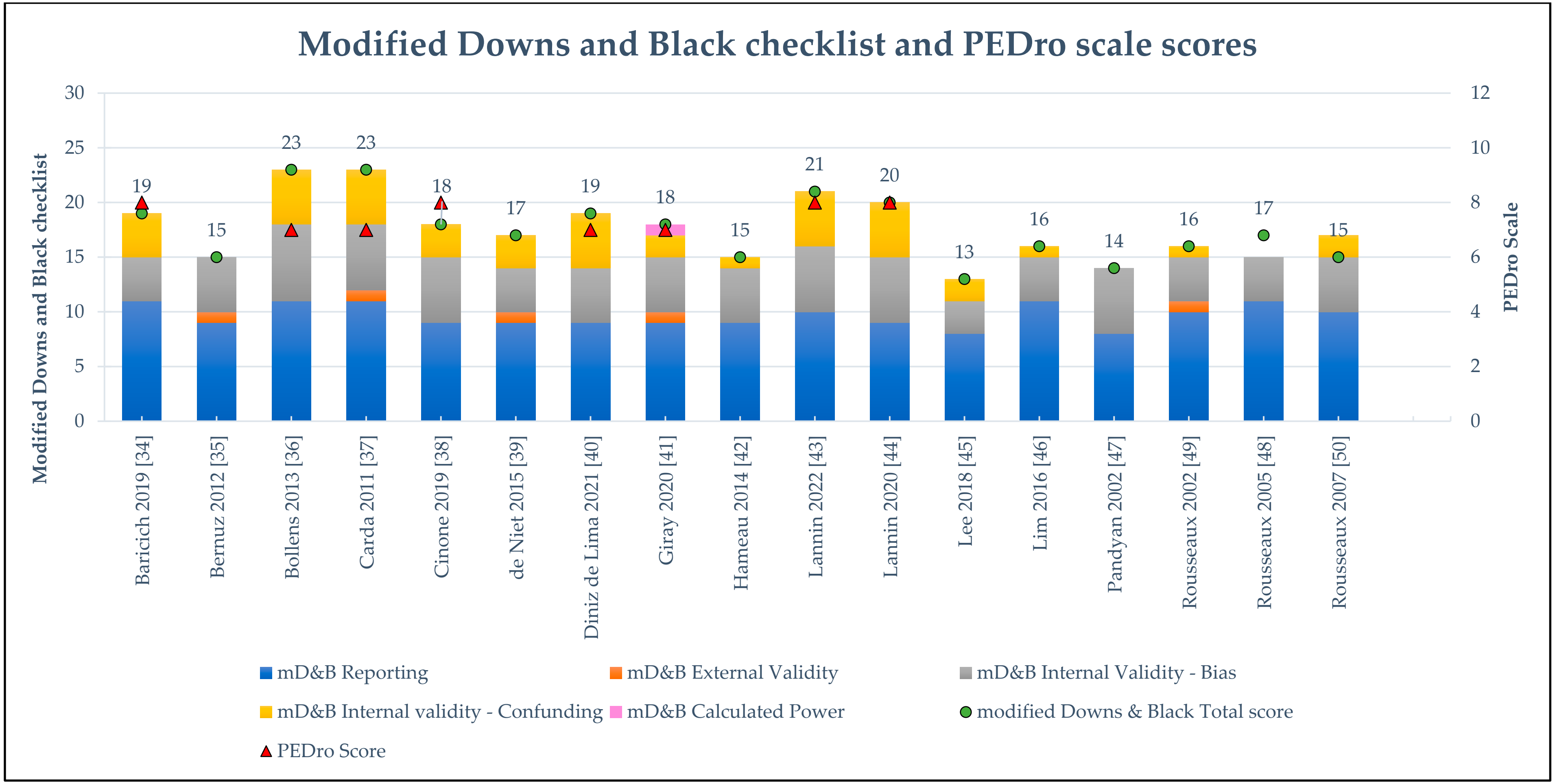

Supplementary File 4. Methodological quality assessment modified Downs and Black checklist and PEDro scale (n = 17).
