# Supplementary material for: The Impact of Strength Changes on Active Function Following Botulinum Neurotoxin-A (BoNT-A): A Systematic Review
Source: Toxins (Basel). 2025 Jul 23;17(8):362. doi: 10.3390/toxins17080362 (PMC12390172; doi:10.3390/toxins17080362)
Supplement: Supplementary file 1 [file toxins-17-00362-s001.zip › toxins-3699136-supplementary/toxins-3699136 Supplementary File 5 - Round 2 Revised.pdf]

Supplementary File 5: Botulinum neurotoxin-A type, dose, dilution, and adjunctive therapies (n = 17).

Author	Treatment arm		BoNT-A: Type, Mean/Median dose (SD), (Range) [IQR], dilution, dose per muscle group	Adjunctive therapies or concurrent therapies
Baricich 2019 [34]	Group 1	ES of injected muscles + ES of TA	(Botox®) (50 - 120) U, 100 U diluted with 2 mL saline 0.9%<br>Individually tailored injection regimes.	ES (0.2 ms, 20 Hz) to the TA muscle (x1 PT session 60 mins/day) in first 1/52.
	Group 2	ES of injected muscles		ES (0.2 ms, 4 Hz) on injected muscles immediately post injection.<br>x1 PT session 60 min/day, calf stretching, 20 min, gait training, aerobic + strengthening exercises.
Bernuz 2012 [35]	Single arm		(Botox®) 200 U distributed in 2 points at each site to RF.	N/A
Bollens 2013 [36]	Group 1	TNN	NA	PT remained unchanged.
	Group 2	BoNT-A control group	100 UI/2 mL dilution<br>Sol 200 UI; TP 125 UI; FHL 75 UI	All participants but 2 (1 patient in each group) received regular PT.
Carda 2011 [37]	Group 1	Casting (1/52) + BoNT-A	(Xeomin®) 100 (50- 140) IU, diluted 2 mL saline 0.9%<br>BB 250 U; BRA 200 U; FCR 150 U; Pt 150 U; FDP 200 U; FPL 50U.	All: 1/52 post injection, 30 mins of gait training + 20 mins PF muscle stretching, daily for 1/52 under guidance of senior PT<br>Casting: Below-knee fibreglass casts applied ankle in neutral supination–pronation and dorsiflexion.
	Group 2	Taping (5 days) + BoNT-A		Taping: 5 days, adjusted daily by a trained PT
	Group 3	Stretching + BoNT-A		Stretching: 30 mins x2/day for 1/52, guided by PT
Cinone 2019 [38]	Experimental	Combined BoNT-A + isokinetic ankle DF training	Onabotulinumtoxin A<br>TS 107.14 (19.70) U; GN - Med 57.14 (18.29) U; GN- Lat 53.33 (14.71); Sol 47.5 (14.33) U	Isokinetic ankle DF training, 4/52, 5 days/week, total 20 sessions.
	Control	BoNT-A alone	Onabotulinumtoxin A<br>TS 120.70 (18.62) U;<br>GN - Med 60.28 (16.50) U, GN- Lat 52.24 (10.04) U;<br>Sol - 53.50 (14.31) U	N/A
de Niet 2015 [39]	Single arm		(Dysport®) 500–750 MU, in 5 mL saline 0.9%.	Twice daily, 10 min calf stretching (with knees flexed and extended) for 18/52.
Diniz de Lima 2021 [40]	Experimental	BoNT-A then Saline + rehabilitation	(Prosigne®) 100 U 2 mL 0.9% sterile sodium chloride dilution, x4 intramuscular vials of 2 mL, 0.9% total of 400 IU<br>AM (100 U) TS 100 U; (¼ in head of each GN, 1/2 in Sol)	Individualised PT (i.e., strengthening/ stretching, proprioception, postural control, gait training, balance), stretching (Add, TS, QUADS, HS, GM) exercises once per day, x3 p/w sessions: x 3 sets 45 seconds of stretching + 15-sec interval training (Add, TS, QUAD, HS, GM).
	Control	Saline then BoNT-A + rehaibilitation	x4 intramuscular vials of 2 mL, 0.9% saline	
Giray 2020 [41]	Experimental	BoNT-A + Lycra sleeve plus rehabilitation	Onabotulinumtoxin A<br>BB 60 IU; BRA 50 IU; PT 30 IU; FCR 30 IU; FCU 30 IU; FDS 30 IU; FDP 30 IU; FPL 20 IU; PQ 20 IU	Lycra sleeve: 8 hr./day, 5 days p/w, 3/52.

Supplementary File 5. Botulinum neurotoxin-A type, dose, dilution, and Adjunctive therapies (n = 17)

			Injected under ultrasound and ES-guidance.	Therapy: passive, active and active-assistive range-of-motion, stretching exercises for shoulder, elbow, wrist, facilitation, and inhibition techniques, neuromuscular ES, strengthening for affected UL + OT 2 hrs./day 5 days p/w.
	Control	BoNT-A + Only rehabilitation control group		Therapy: passive, active and active-assistive range-of-motion, stretching exercises for shoulder, elbow, wrist, facilitation, and inhibition techniques, neuromuscular ES, strengthening for affected UL + OT 2 hrs./day 5 days p/w.
Hameau 2014 [42]	Single arm		Onabotulinumtoxin A (Botox®), Dose - NR.	N/A
Lannin 2020 [44]	Experimental	BoNT-A + casting + movement training	(Botox®) max 600 U, 100 U (max volume per site = 0.5-1.0 mL). Muscles crossing the wrist.	BoNT-A + 2/52 serial casting applied to place the wrist in maximal extension for 2/52, + 10/52 of movement training (ES and progressive resistance training). 60 mins per day, 7 days p/w, 10/52 (~70 hrs. total).
	Control	BoNT-A + HEP		Usual care (not stipulated)
Lannin 2022 [43]	Experimental	BoNT-A + 2/52 of serial casting + 10/52 movement training	(Botox®) max 600 U, 100 U (max volume per site = 0.5-1.0 mL). Muscles crossing the wrist.<br>FCR E: 49(19), C: 47(16); FCU E: 48(19) C: 50(18); FDS 52(18) C: 53(21) FDP E: 47(15) C: 51 (20); FPL E: 32(21) C: 38 (22); ECRL E: 34(22) C: 20(14) (supplied as supplementary material)	BoNT-A + 2/52 serial casting followed + 10/52 of movement training. 2/52 serial casting in max wrist extension + 10/52 of movement training. 60 mins per day, 7 days p/w, for 10/52 (~70 hrs. total).
	Control	BoNT-A + HEP		HEP + x1 follow-up phone call to encourage independence. HEP (7 stretches, 8 arm, hand exercises). Participants did not receive other UL rehabilitation.
Lee 2018 [45]	Single arm	BoNT-A + ES	Onabotulinumtoxin A (Botox®), max 360 U, 100 U diluted 2 mL saline (5 U/0.1 mL). Individual patient injection dosages reported in Table 3. Muscles selected according to individual's spasticity.<br>≥ 1 elbow flexor muscles (BB, BR, BRA).	2/52 post BoNT-A to finger +/- wrist flexors, ES of FE + wrist brace for 4/52 (5 days p/w; 30 mins).
Lim 2016 [46]	Subacute Group		(Botox®) 200 U, 100 U vial diluted 2 mL normal saline.<br>Injected muscles and appropriate doses chosen for the individual muscle.<br>≥ 1 EF muscles (BB, BR, BRA) + ≥ 1 WF muscles (FCR, FCU) or ≥ 1 FF muscles (FDP, FDS, FPL).	Inpatient or outpatient rehabilitation treatment 2 days p/w
	Chronic group			
Pandyan 2002 [47]	Single arm		(Botox®) 100-190 MU, dilution of 100 MU in 2.5ml. (BB 70 U); BR 56.5 U; EDL 83.3 U.<br>(1 MU of Botox® equivalent to 3 MU of (Dysport®)<br>(Dysport®) dilution 500 MU in 2.5 mL of saline 100-500 MU	N/A
Rousseaux 2002 [49]	Single Arm		(Botox®) 200 - 300 U, diluted 100 U/ml.	PT/OT kept constant throughout study (5/12).
Rousseaux 2005 [48]	Single Arm		(Botox®) 300 U; Sol 123.8 U; GN 78.9 U; TP 47.8 U; FDL 52.7 U; FHL 52.7 U; TA 54.3 U.	PT was kept as constant throughout the study, 5/12
Rousseaux 2007 [50]	Single Arm		(Botox®) 400 U, 50 U/ml. Sol + GN 160 U, TP 40 U, FDL 40 U, AL/AM 80-100 U. Individually tailored injection regimes.	PT was kept as constant throughout the study, 5/12.

AM – Adductor Magnus; BB - Biceps Brachii; BF – Biceps Femoris; BI – Brain injury; BoNT- A- Botulinum-toxin A;BRA – Brachialis; C – Control group; D – Deltoid; DF – Dorsiflexion; EDL – Extensor Digitorum Longus; EF – Elbow Flexors; ES – E-Stims; FCR – Flexor Carpi Radialis; FCU – Flexor Carpi Ulnaris; FDL – Flexor Digitorum Longus; FDP – Flexor Digitorum Profundus; FDS – Flexor Digitorum Superficialis; FE – Finger Extension; FF – Finger flexion; FHL – Flexor Hallucis Longus; FPL – Flexor Pollicis Longus; GM – Gluteus Maximus; GN – Gastrocnemius; HEP – Home exercise program; HS – Hamstrings; Hz – Hertz; IQR – Interquartile range; IU – international unit; Lat – lateral; LL – Lower limb; med – medial; Mins – Minutes; mL - milliliters; MU -

Supplementary File 5. Botulinum neurotoxin-A type, dose, dilution, and Adjunctive therapies (n = 17)

Mouse units; N/A – Not Applicable; NR – Not Reported; OT – Occupational Therapy/Occupational Therapist; p/w – per week; PQ – Pronator Quadratus; Pt – Pronator Teres; PT-Physiotherapy/Physiotherapist; QUADS – Quadriceps; RF – Rectus Femoris; SD – Standard deviation; Sol – Soleus; TA – Tibialis Anterior; TNN – Tibial nerve neurotomy; TP – Tibialis Posterior; TS – Triceps Surae; U – units; UL – Upper Limb; WF – Wrist Flexion.

Supplementary File 5. Botulinum neurotoxin-A type, dose, dilution, and Adjunctive therapies (n = 17)
